# Prokaryotic gasdermins: ancestors of eukaryotic counterparts direct the pyroptosis and cell fates

**DOI:** 10.1038/s41392-022-01005-y

**Published:** 2022-05-07

**Authors:** Yingying Liu, Chen Zhang, Min Wu

**Affiliations:** 1 Department of Biomedical Sciences, School of Medicine and Health Sciences, University of North Dakota, Grand Forks, ND USA; 2 Laboratory of Microbiology, Wageningen University & Research, Stippeneng 4, 6708 WE Wageningen, The Netherlands; 3 Biotechnology Research Institute, Chinese Academy of Agricultural Sciences, Beijing, 100081 People’s Republic of China

**Keywords:** Innate immunity, Structural biology, Structural biology

Recently, the role of bacterial gasdermin (bGSDM) has been biochemically characterized and published in *Science* by Johnson et al., which bridges the gap in functional understanding of GSDM between prokaryotes and eukaryotes, and also suggests the evolutionary conservation of GSDM-mediated pyroptosis, leading to cell death.^1^ In addition, the identified bGSDM adds unprecedented insights into understanding of the bacterial immunity.

Gasdermin D (GSDMD), as one of the six gasdermin proteins (from GSDMA to GSDME, pejvakin) encoded by human genomes, remained unknown before 2015. In 2015, two research teams independently discovered that the human gasdermin D (GSDMD) switched to active from autoinhibition via the cleavage of its C-terminal domain (CTD) by inflammatory caspases and the other regulatory factors, cleaved N-terminal domain (NTD), was reorganized into oligomers spanning across the membrane to form gasdermin pores, which resulted in the secretion of mature IL-1β and lead to cell death.^2–4^ Sequence analyses revealed that there existed a clade of 50 bGSDM homologs distinctive to the eukaryotic counterparts. On the structural basis of bGSDM from *Bradyrhizobium tropiciagri* and *Vitiosangium sp*., the overall architecture, particularly the NTD, exhibited strong homology consists of twisted central antiparallel β sheet, linking helices and strands.^1^ Although the structural study of bGSDM revealed the absence of large alpha-helical CTD that is required to maintain the autoinhibition of mammalian gasdermins, the bGSDM recruited a molecule with the same configuration to achieve the inactivation of gasdermins.^1^ The atomic model of bGSDM deciphered that the protrusion from cysteine C3 side-chain in bGSDM from *Bradyrhizobium* occupied a hydrophobic tunnel, which can be capped by a residue, F25 at the C-terminal domain and this feature was verified as palmitoylation.^1^ The cysteine is a conserved site in most bacterial and some fungal gasdermins, and is critical to the palmitoylation of bGSDM, which was assumed to stabilize the inactive state of bGSDM. The residues alongside the hydrophobic tunnel could be substantially reorganized during bGSDM activation inferred from modeling as Johnson simulated.^1^

**Fig. 1:**
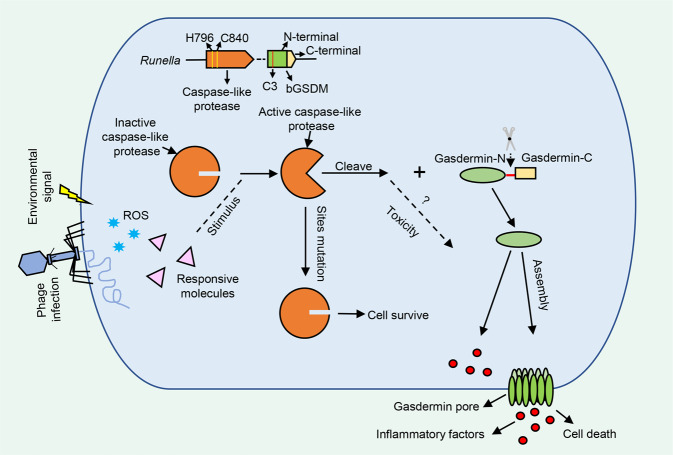
Mechanism of bGSDM-mediated cell death. On recognition of external stimuli, such as phage and environmental matters, the caspase-like proteases are activated. Co-expression of bGSDM and their cognate proteases leads to bacterial growth arrest and membrane permeabilization. bGSDM palmitoylation (C3 in red line) is essential for particularly strong toxicity in *Runella* system. Mutations of the catalytical residues (H796 and C840 in yellow lines) in caspase-like protease abolished cellular toxicity and blocked cell death. The autoinhibited bGSDM was cleaved by caspase-like protease into N- and C-terminal domains. The active N-terminal domains are oligomerized to form bGSDM pore spanning the membrane that can permeabilize liposomes and thereby releasing inflammatory factors, and consequently leads to cell lysis

GSDM activation specifically depends on the cleavage by caspases in eukaryotic cells.^4,5^ In most bacterial cases, the caspase-like protease was clustered together with bGSDM shown in Fig. 1. Furthermore, the bGSDM-caspase system was found widely distributed in bacteria and archaea. Intriguingly, the bGSDMs in some cases were accompanied by well-identified bacterial immune systems, such as CRISPR-cas and abortive infection systems. Accordingly, Johnson et al., assumed the bGSDM-caspase could have the potential for bacterial host defense against phage infection. A four-gene operon containing the bGSDM gene, from *Lysobacter enzymogenes*, was heterologously expressed, in which the presence of bGSDM can substantially protect the host *Escherichia coli* from infection by coliphages T4, T5, and T6.^1^

A series of mechanistic studies were carried out to investigate the molecular details. The mutation of S293A of the second trypsin-like protease in bGSDM system from *Lysobacter* disrupted the antiphage defense of the recipient.^1^ In addition, the expression of bGSDM system from *Runella*, which required palmitoylation, introduced escalated cellular toxicity without phage infection (Fig. 1). Additional propidium iodide (PI) staining analysis presented a clear image of destroyed membrane and stopped cell division of the host. In Supplementary studies, more site-directed mutagenesis of caspase-like protease in *Runella* abolished all cellular toxicity generation, indicating the activation of bGSDM system enabled by proteolytic cleavage. In vitro experiments verified that *Runella* bGSDM species were produced after the incubation of protease and bGSDM, in which the targeting cleavage site of bGSDM occurred in a loop after P1 residue L247. Further, C-terminal truncation of bGSDMs in *Runella*, *Bradyrhizobium*, or *Vitiosangium* resulted in the cell death, unveiling an essential role of C-terminal in bGSDM autoinhibition. In addition to P1 L247 mutation, sites-mutagenesis, including P1’ glycine, P2, P3, P4 and P3’, prohibited proteolysis. Meanwhile, disruption of P1 and P1’ positions abolished in vivo toxicity.^1^ Similar to the release of mature IL-1β by mammalian GSDMD,^4^ the abridged bGSDM from *Runella* permeabilized liposomes and released cytoplastic molecules to outside the membrane.^4^ In general, the neighboring caspase-like protease is indispensable for bGSDM activation, but mutations involved in bGSDM palmitoylation cannot avoid liposome leakage and puncta formation. More of interests, the pore architectures of bGSDM are diverse and different from mammalian counterparts in size, which might be shaped by the release of specific internal contents.

Revelation of functional conservativeness of gasdermins across the lifeforms from prokaryotes to eukaryotes represents a significant advance to reassess and broaden the understanding of bacterial immunity. The concomitant responses of the host alongside bGSDM activation, such as increased cytotoxic activity, were observed but the underlying mechanisms remain elusive in diverse living environments and other activation states. In particular, several important questions are unclear: considering the diversity and challenge of inhabiting environments of prokaryotes, how do bGSDMs coordinate with their neighboring immune systems to adapt or reject foreign invaders, such as phage infection, is still unknown. Furthermore, bGSDMs are cleaved by caspase-like proteases under normal conditions, but would this cleavage activity have any biological significance under phage infections or abiotic stresses is largely undisclosed.

Taken together, this pioneering work of Johnson et al., demonstrates the evolutionary continuity of gasdermins from prokaryotes to eukaryotes. Undoubtedly, the discovery of bacterial GSDMs inspires researchers to explore the presence and roles of bacterial inflammasomes, predict homology to mammalian, such as nucleotide-binding domain and leucine-rich repeat receptors (NLRs), the absent in melanoma 2-like receptors (ALRs), pyrin, and so on. Moreover, functional assessment of inflammasomes from invertebrates, fungi and bacteria will be a great contribution to the field of comparative immunology. Importantly, these findings may instigate ideas on studying gasdermin-mediated pyroptosis and pharmaceutical development targeting pyroptosis-related diseases. Meanwhile, multi-omics’ data analyses become necessary to decode the genetic information of culture-independent bacteria and archaea. Built on the recent discovery of the cGAS-like pathway in bacteria, this intriguing work by Johnson et al., further expands the boundary of the bacterial immunity systems and adds evidence of ancestral origins of the mammalian immune systems from prokaryotes.
